# Health Practices and Mortality in Japan: Combined Effects of Smoking, Drinking, Walking and Body Mass Index in the Miyagi Cohort Study

**DOI:** 10.2188/jea.14.S39

**Published:** 2005-03-18

**Authors:** Yoshitaka Tsubono, Yayoi Koizumi, Naoki Nakaya, Kazuki Fujita, Hideko Takahashi, Atsushi Hozawa, Yoko Suzuki, Shinichi Kuriyama, Ichiro Tsuji, Akira Fukao, Shigeru Hisamichi

**Affiliations:** 1 Division of Epidemiology, Department of Public Health and Forensic Medicine, Tohoku University Graduate School of Medicine.; 2 Department of Public Health, Yamagata University School of Medicine.

**Keywords:** smoking, alcohol, walking, body mass index, mortality

## Abstract

BACKGROUND: Evidence is limited regarding the association between the combinations of multiple health practices and mortality.

METHODS: In 1990, 28,333 men and women in Miyagi Prefecture in rural northern Japan (40-64 year of age) completed a self-administered questionnaire. A lifestyle score was calculated by adding the number of high-risk practices (smoking, consuming ≥22.8 g alcohol/d, walking <1 hr/d, body mass index **<**18.5 or ≥30.0). Cox regression was used to estimate relative risk (RR) of mortality according to the lifestyle score, with adjustment for age, education, marital status, past history of diseases, and dietary variables. During 11 years of follow-up, 1,200 subjects had died.

RESULTS: We observed linear increase in risk of death associated with increasing number of high-risk practices: compared with men who had no high-risk practices, multivariate RRs for men who had 1 to 4 practices were 1.20, 1.66, 1.94, and 3.96, respectively (P for trend<0.001), and corresponding RRs for women were 1.31, 2.14, 3.98, 5.56, respectively (P for trend<0.001). A unit increase in the number of high-risk practices corresponded to being 2.8 and 4.8 years older for men and women, respectively.

CONCLUSONS: In this prospective cohort study of middle-aged men and women in rural Japan, a larger number of high-risk practices was associated with linear increase in risk of all-cause mortality.

Various studies have shown that smoking,^1^ high alcohol consumption,^2^ physical inactivity,^3^ and obesity or underweight,^4^ are associated with increased risk of all-cause mortality. However, only a limited number of studies have examined the combined effects of these factors on mortality.^5^^-^^8^ Furthermore, the studies used relatively small number of subjects (<7,000) and small number of cases of death (<800).

To examine how the risk of all-cause mortality increases as people have more of the four high-risk health practices (smoking, high alcohol consumption, physical inactivity indicated by shorter duration of walking, and obesity or underweight), we conducted a population-based prospective cohort study in rural northern Japan, which involved 28,333 subjects and 1,200 cases of death ascertained during 11 years of follow-up.

## METHODS

### Study Cohort

We have reported the design of this prospective cohort study in detail elsewhere.^9^ Briefly, from June through August 1990, we delivered a self-administered questionnaire on various health habits to 51,921 subjects (25,279 men and 26,642 women) who were 40-64 years of age and lived in 14 municipalities of Miyagi Prefecture in northern Japan. The questionnaires were delivered to and collected from the subjects’ residences by members of health promotion committees appointed by the municipal governments. Usable questionnaires were returned from 47,605 subjects (22,836 men and 24,769 women), yielding a response rate of 91.7%.

The study protocol was approved by the institutional review board of Tohoku University Graduate School of Medicine. We considered the return of the self-administered questionnaires signed by the subjects to imply their consent to participate in the study.

### Exposure Data

We used information on four health practices (smoking, alcohol consumption, walking, and body mass index) collected by the questionnaire. The accompanying papers described in detail how the questionnaire asked about the four practices.^1^^-^^4^ We dichotomized each variable and assigned 1 or 0 point to potentially unhealthy or healthy habits, respectively (Table 1). We then summed these points and determined the number of high-risk practices for each subject. The total score ranged 0-4 points, with higher scores indicating lifestyles with higher risk.

**Table 1.** Scoring classifications of health practices.

	0 point (Low Risk)	1 point (High Risk)
Smoking	Never	Current
Drinking	< 22.8 g/day	22.8 g/day≤
Walking	1 hr/day≤	<1 hr/day
Body Mass Index	18.5≤BMI<30.0	<18.5, 30.0≤

We determined the cut-off points for dichotomizing the variables based on the observations in the accompanying papers which examined the association between each of the four factors and mortality from all causes.^1^^-^^4^ Specifically, we considered those categories among each variable that were associated with increased mortality as “unhealthy” (assigning 1 point), and the other categories as “healthy” (assigning 0 point).

### Follow-up

Of 47,605 subjects who responded to the questionnaire, we excluded 1,522 subjects who indicated that they had prior histories of cancer (n=561), stroke (n=379), or myocardial infarction (n=582). We also excluded 539 subjects who had prevalent cancer that were ascertained by record linkage to the population-based cancer registry covering the study area.^10^ We further excluded subjects who had incomplete responses for any of the four health practices (n=11,983), and subjects who were past smoker (n=3,342) and past drinker (n=1,386). Consequently, 28,333 subjects (13,987 men and 14,346 women) with 1,200 deaths (805 men and 395 women) were included in this analysis.

We followed up vital and residential status of subjects from June 1, 1990, through March 31, 2001. For this follow-up, we established the Follow-up Committee that was consisted of Miyagi Cancer Society; Community Health Division of all 14 municipalities; Department of Health and Welfare, Miyagi Prefectural Government; and Division of Epidemiology, Tohoku University Graduate School of Medicine. The Committee periodically reviewed the Residential Registration Record of each municipality. With this review, we identified the subjects who either died or emigrated during observation. For both decedents and emigrants, we recorded the date of death or emigration. For decedents, we investigated cause of death by reviewing the death certificates of the subjects at Public Health Centers of the study area. The underlying cause of death was coded according to International Classification of Diseases, the Ninth Revision (ICD-9). We discontinued follow-up of subjects who emigrated from the study municipalities because of logistical limitations.

We counted person-years of follow-up for each subject from June 1, 1990, until the date of death, the date of emigration outside the study districts, or the end of the study period (March 31, 2001), whichever occurred first. A total of 293,521 person-years accrued. There were 1,389 subjects (4.9% of the analysis cohort) who emigrated from the study municipalities and were lost to follow-up.

### Statistical Analysis

We used Cox proportional-hazards regression to estimate relative risk (RR) and 95% confidence interval (CI) of all-cause mortality according to the number of high-risk health practices and to adjust for potentially confounding variables, using the PHREG procedure on SAS^®^ version 8.2 statistical software package (SAS Inc., Cary, NC, USA). We conducted all analyses separately for men and women.

We considered following variables as potential confounders: age in years; education (up to 15 years of age, 16-18, or 19 years or older); marital status (whether or not living with spouse at baseline); past histories of hypertension, renal diseases, liver diseases, diabetes mellitus, peptic ulcers, or tuberculosis; consumption frequencies of green vegetables and oranges (almost daily, 3-4 times per week, 1-2 times per week, or 1-2 times per month or less often).

We estimated how a unit increase in the number of high-risk health practices corresponded to an increase in the years of age of the subjects.^11^ Specifically, a regression coefficient for the number of high-risk practices entered as a continuous variable into Cox regression model was divided by a regression coefficient for age entered as a continuous variable into the same model.

We repeated all analyses after excluding 194 subjects (124 men and 70 women) who died during the first three years of follow-up. We also conducted stratified analyses according to the categories of covariates included in the multivariate analyses to examine whether the association between the number of high-risk practices and all-cause mortality was modified by these variables. P values for tests of linear trends were estimated using the number of high-risk practices as a continuous variable. All P values were two-tailed.

## RESULTS

**Figure 1.**
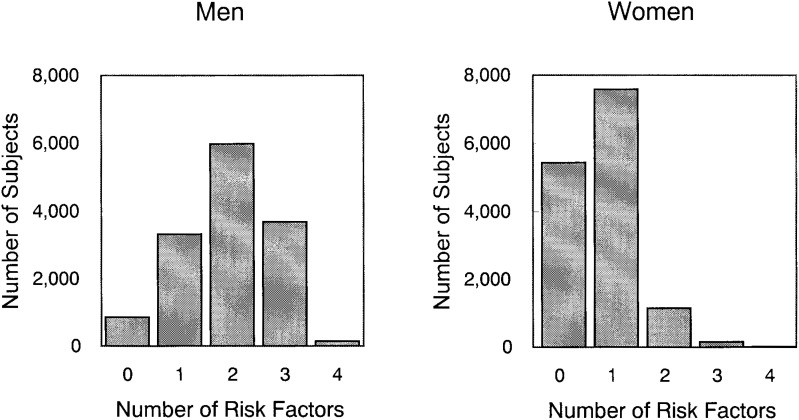
Distribution for the number of risk factors for men and women.

Figure 1 shows the distribution of the number of high-risk health practices by sex. For men, the number approximates normal distribution with the mode of 2 points. For women, it shows skewed distribution with the mode of 1 point.

Table 2 compares the characteristics of subjects according to the number of high-risk practices. The men with higher number of high-risk practices tended to be younger, more educated, consume green vegetables and oranges less frequently, and have past histories of hypertension, liver disease, diabetes, and peptic ulcer. The women with higher number of high-risk practices tended to be younger, consume green vegetables and oranges less frequently, be less likely to live with spouse, and have past histories of liver disease and peptic ulcer.

**Table 2.** Characteristics of subjects according to the number of high-risk health practices.

	Number of High-risk Health Practices, Men					Number of High-risk Health Practices, Women				
	
	0	1	2	3	4	0	1	2	3	4
No. of subjects	857	3,323	5,980	3,683	144	5,436	7,588	1,151	161	10
Mean age (SD)	52.1 (7.3)	51.2 (7.4)	50.6 (7.5)	49.7 (7.5)	49.8 (7.9)	51.8 (7.2)	50.5 (7.5)	50.0 (7.4)	48.8 (7.2)	46.7 (6.2)
Body Mass Index (%)										
<18.5	0.0	0.5	0.9	3.7	58.3	0.0	2.0	18.4	18.0	70.0
18.5-24.9	71.0	72.5	74.8	70.4	0.0	71.8	68.1	45.3	47.2	0.0
25≤	29.0	27.0	24.3	26.0	41.7	28.2	29.9	36.3	34.8	30.0
Education (%)										
≤15	45.5	40.8	39.8	32.8	29.3	38.3	34.9	36.4	38.2	60.0
16-18	43.0	44.8	46.5	49.8	53.6	48.2	49.5	48.9	49.3	40.0
19≤	11.5	14.4	13.7	17.4	17.1	13.5	15.6	14.7	12.5	0.0
Living with spouse (%)										
Yes	90.8	92.5	92.3	92.9	92.2	89.0	87.5	82.3	81.0	70.0
No	9.2	7.5	7.7	7.1	7.8	11.0	12.5	17.7	19.0	30.0
Past history (%)										
Hypertension	11.8	13.5	16.6	19.5	22.9	17.7	19.0	20.3	17.4	30.0
Renal diseases	4.0	2.7	3.0	3.1	5.6	3.7	4.3	4.0	6.2	10.0
Liver diseases	4.6	4.5	5.7	7.0	11.8	3.4	3.4	4.3	5.0	0.0
Diabetes mellitus	3.6	4.4	4.2	5.9	5.6	2.3	3.0	3.4	1.2	0.0
Peptic ulcers	15.4	17.7	20.6	21.6	23.6	8.2	9.7	11.0	14.9	0.0
Tuberculosis	2.8	2.6	3.0	3.1	6.9	2.1	2.5	4.3	3.1	0.0
Green vegetables (%)										
≤1-2/month	10.8	13.2	15.6	17.7	16.2	6.9	7.4	11.1	14.5	20.0
1-2/week	31.5	34.1	35.3	38.7	40.4	26.6	31.0	31.9	40.7	40.0
3-4/week	32.2	32.2	29.0	27.6	25.0	36.9	36.6	33.7	31.7	30.0
Everyday	26.5	20.5	20.1	16.0	18.4	29.6	25.0	23.3	13.1	10.0
Oranges (%)										
≤1-2/month	20.7	23.5	31.8	36.0	41.2	14.1	13.5	19.5	31.9	44.4
1-2/week	27.1	29.6	29.8	32.3	26.7	18.9	20.3	21.7	20.1	0.0
3-4/week	24.7	25.2	22.0	19.5	20.6	27.1	27.9	26.4	26.4	22.2
Everyday	27.5	21.7	16.4	12.2	11.5	39.9	38.3	32.4	21.6	33.4
Smoking status (%)										
Never	100.0	51.0	13.9	6.7	0.0	100.0	95.5	51.2	4.7	0.0
1-19 cigarrets/day	0.0	13.4	21.9	22.0	17.0	0.0	3.5	34.7	54.0	44.4
20≤ cigarrets/day	0.0	35.6	64.2	77.3	83.0	0.0	1.0	14.1	41.3	55.6
Alcohol drinking (%)										
22.8≤	49.2	62.8	84.6	97.8	100.0	20.8	24.5	46.9	78.3	100.0
<22.8	50.8	37.2	15.4	2.2	0.0	79.2	75.5	53.1	21.7	0.0
Walking (%)										
1 hr/day≤	100.0	69.8	51.2	3.1	0.0	100.0	9.5	9.9	2.5	0.0
<1 hr/day	0.0	30.2	48.8	96.9	100.0	0.0	90.5	90.1	97.5	100.0

SD: standard deviation

For both men and women, we observed significant, linear increase in age-adjusted risk of all-cause mortality associated with the larger number of high-risk health practices (Table 3). A unit increase in high-risk practices corresponded to being 2.8 years older for men and 4.8 years for women. These results remained basically unchanged with multivariate adjustment or with the exclusion of death occurring within the first 3 years of follow-up. When the women with 3 or 4 high-risk practices were combined into one group, RRs (95% CIs) of all-cause mortality for this group of women as compared with women with no such practice were 4.34 (2.40-7.85) with adjustment for age only, 4.08 (2.24-7.42) with multivariate adjustment, and 4.36 (2.26-8.40) with multivariate adjustment and with the exclusion of subjects who dies within the first 3 years of follow-up.

**Table 3.** Relative risk (RR) of all-cause mortality according to the number of high-risk health practices in men and women.^†^

	Number of High-risk Health Practices					P for trend

	0	1	2	3	4	
Men						
Person-years	8,962	34,555	61,541	37,522	1,445	
No. of death	35	152	358	241	19	
Age-adjusted RR	1.0	1.21 (0.84 - 1.75)	1.69 (1.19 - 2.39)	2.04 (1.43 - 2.90)	4.24 (2.43 - 7.41)	<0.001
Multivariate RR1	1.0	1.20 (0.83 - 1.74)	1.66 (1.17 - 2.35)	1.94 (1.36 - 2.78)	3.96 (2.26 - 6.95)	<0.001
Multivariate RR2	1.0	1.22 (0.82 - 1.83)	1.76 (1.20 - 2.57)	1.95 (1.32 - 2.89)	3.62 (1.91 - 6.88)	<0.001

Women						
Person-years	57,162	78,909	11,762	1,565	98	
No. of death	124	207	52	11	1	
Age adjusted RR	1.0	1.32 (1.06 - 1.65)	2.36 (1.71 - 3.26)	4.18 (2.25 - 7.74)	7.57 (1.06 - 54.25)	<0.001
Multivariate RR1	1.0	1.31 (1.04 - 1.63)	2.14 (1.54 - 2.97)	3.98 (2.14 - 7.42)	5.56 (0.77 - 40.29)	<0.001
Multivariate RR2	1.0	1.32 (1.03 - 1.68)	2.21 (1.54 - 3.17)	4.16 (2.09 - 8.27)	7.68 (1.06 - 55.63)	<0.001

† : Adjusted for age in years; education (up to 15 years of age, 16-18, or 19 years or older); marital status at baseline (whether or not living with spouse; past history of hypertension, renal diseases, liver diseases, diabetes mellitus, peptic ulcers, or tuberculosis; consumption frequencies of green vegetables and oranges (almost daily, 3-4 times per week, 1-2 times per week, or 1-2 times per month or less often). Multivariate RR2 has been estimated with the exclusion of 194 subjects (124 men and 70 women) who died within the first 3 years of follow-up. Numbers in parentheses are 95% confidence intervals.

In multivariate analyses stratified by the consumption frequency of green vegetables, the increased risk of mortality associated with the higher number of risk factors was observed among subjects who consumed green vegetables less often than daily (P for trend<0.001 for both men and women), while it was not apparent among subjects who consumed green vegetables daily (P for trend=0.097 for men and 0.422 for women). We observed no differential findings in stratified analyses by other covariates.

## DISCUSSION

This population-based prospective cohort study of middle-aged men and women in rural northern Japan examined the combined effects of four health practices (smoking, alcohol consumption, body build, and walking) on mortality from all causes. A larger number of high-risk practices (current smoking, high alcohol consumption, short duration of walking, and underweight or obesity) was associated with linear increase in risk. The subjects with the largest number of high-risk practices (4 for men and 3-4 for women) had four-fold risk of death as compared with the subjects with no such practice, and a unit increase in high-risk practices corresponded to being 2.8 years older for men and 4.8 years older for women.

Table 4 summarizes five studies, including this study, examining the effects of combined health practices on all-cause mortality. The studies were conducted in Japan,^6^^,^^7^ U.S.,^5^ and Europe.^8^ The sample sizes ranged 1,281 through 28,333. The number of health practices examined ranged 3 through 7. All of the five studies included smoking and measures of physical activity (such as walking and exercise), and all but one studies included measures of body builds (BMI or obesity defined in relation to weight relative to height). In contrast, only three studies included duration of sleeping, and only two studies included dietary variables.

**Table 4.** Summary of studies examining the effects of combined health practices on mortality from all causes.

Author Year	Country Age Sex	Subjects Death Follow-up (yrs)	Health Practice	Low Risk	High Risk	No. of Health Practice	Multivariate Relative Risk (95% CI)			
Breslow^5^ 1993	US (Alameda, CA)	6,928	Smoking	Never	Current, Ex	Low Risk				
	20-	719	Drinking	<5 drinks at a time	≥5 drinks at a time	0-3	1.00	(Reference)		
	Men and women	9	Physical activity	High	Low	4-5	0.63	(0.53 - 0.76)		
			Obesity	No	Yes	6-7	0.45	(0.35 - 0.57)		
			Sleeping	7-8 hr/d	≤6, ≥9 hr/d					
			Eating between meals	No	Yes					
			Eating breakfast	Yes	No					

Tsubono^6^ 1993	Japan (Miyagi)	4,318	Smoking	Never	Current, Ex	Low Risk				
	40-	207	Drinking	< 68.4 g/d	≥ 68.4 g/d, Ex	0-1	1.00	(Reference)		
	Men and women	4	Exercise	≥1 hr/wk	Rarely	2	0.63	(0.38-1.05)		
			BMI	≥21.2	<21.2	3	0.49	(0.30-0.80)		
			Sleeping	7-8 hr/d	≤6, ≥9 hr/d	4	0.27	(0.14-0.50)		
						5	0.14	(0.03-0.61)		

							P for trend = 0.0001			

Morioka^7^ 1996	Japan (Wakayama)	3,048	Smoking	Never, Ex	Current	Low Risk	Men		Women	
	40-79	171	Drinking	≤22.8 g/d	>22.8 g/d	5-4	1.0	(Reference)	1.0	(Reference)
	Men and women	6	Exercise	≥1 hr/wk	Rarely	3	1.0	(NS)	1.2	(NS)
			BMI	18-26	<18, >26	2-0	1.4	(NS)	1.8	(NS)
			Sleeping	7-8 hr/d	≤6, ≥9 hr/d					

Haveman-Nies^8^ 2002	7 European Countries	1,281	Smoking	Never, Ex (>15 yrs)	Current, Ex (≤15 yrs)	High Risk	Men		Women	
	70-75	472	Physical activity	High	Low	0	1.0	(Reference)	1.0	(Reference)
	Men and women	11	Diet quality	High	Low	1	1.2-2.1		1.3-1.8	
						2	1.7-2.8		2.2-3.1	
						3	3.5		3.9	

Present study 2003	Japan (Miyagi)	28,333	Smoking	Never	Current	High Risk	Men		Women	
	40-64	1,200	Drinking	< 22.8 g/d	≥22.8 g/d	0	1.00	(Reference)	1.00	(Reference)
	Men and women	11	Walking	≥1 hr/d	<1 hr/d	1	1.20	(0.83-1.74)	1.31	(1.04-1.63)
			BMI	18.5-30	<18.5, ≥30	2	1.66	(1.17-2.35)	2.14	(1.54-2.97)
						3	1.94	(1.36-2.78)	3.98	(2.14-7.42)
						4	3.96	(2.26-6.95)	5.56	(0.77-40.29)

							P for trend < 0.001		P for trend < 0.001	

CI: confidence interval

NS: not significant

The studies used various criteria to dichotomize health practices into high risk and low risk categories. For instance, past smokers were included into high risk category,^5^^,^^6^ low risk category,^7^ or both categories depending on years since quitting,^8^ or excluded from analyses (the present study), while current smokers and never smokers were constantly included into high and low risk categories, respectively. Nevertheless, all studies are consistent in showing linear increase in risk of mortality associated with the increasing number of high-risk practices.

Our study has several strengths. First, the study involved the largest number of subjects, the longest duration of follow-up, and the largest number of death, as compared with the previous studies.^5^^-^^8^ Second, we included only health practices that were significantly associated with all-cause mortality in our subjects, even when they were considered individually.^1^^-^^4^ This choice of health practices would make our scoring method readily interpretable. Third, we adjusted for potential confounders as much as possible in the analyses, which included age, socioeconomic status (education and marital status), and baseline health status (past history of diseases).

Our study has potential limitations. First, the assessment of health practices was based on the self-administered questionnaire, which would have lead to misclassification of subjects to some extent with regard to the actual risk status. Second, because this is an observational study rather than an intervention study, the observed higher mortality associated with the larger number of high-risk practices does not directly translate into expected mortality reduction when people decrease the number of high-risk practices. Third, because this study is conducted among middle-aged rural Japanese population, the generalizability of our observations to other populations may be limited.
